# Annealing Induced Re-crystallization in CH_3_NH_3_PbI_3−x_Cl_x_ for High Performance Perovskite Solar Cells

**DOI:** 10.1038/srep46724

**Published:** 2017-04-21

**Authors:** Yingguo Yang, Shanglei Feng, Meng Li, Weidong Xu, Guangzhi Yin, Zhaokui Wang, Baoquan Sun, Xingyu Gao

**Affiliations:** 1 Shanghai Institute of Applied Physics, Chinese Academy of Sciences, 2019 Jialuo Road, Shanghai 201800, China; 2 University of Chinese Academy of Sciences, Beijing 100049, China; 3 Jiangsu Key Laboratory for Carbon-Based Functional Materials & Devices, Institute of Functional Nano & Soft Materials (FUNSOM), Soochow University, 199 Ren’ai Road, Suzhou, 215123, China

## Abstract

Using poly(3,4-ethylenedioxythiophene):polystyrene sulfonate (PEDOT:PSS) as hole conductor, a series of inverted planar CH_3_NH_3_PbI_3−x_Cl_x_ perovskite solar cells (PSCs) were fabricated based on perovskite annealed by an improved time-temperature dependent (TTD) procedure in a flowing nitrogen atmosphere for different time. Only after an optimum annealing time, an optimized power conversion efficiency of 14.36% could be achieved. To understand their performance dependence on annealing time, an *in situ* real-time synchrotron-based grazing incidence X-ray diffraction (GIXRD) was used to monitor a step-by-step gradual structure transformation from distinct mainly organic-inorganic hybrid materials into highly ordered CH_3_NH_3_PbI_3_ crystal during annealing. However, a re-crystallization process of perovskite crystal was observed for the first time during such an annealing procedure, which helps to enhance the perovskite crystallization and preferential orientations. The present GIXRD findings could well explain the drops of the open circuit voltage (V_oc_) and the fill factor (FF) during the ramping of temperature as well as the optimized power conversion efficiency achieved after an optimum annealing time. Thus, the present study not only illustrates clearly the decisive roles of post-annealing in the formation of solution-processed perovskite to better understand its formation mechanism, but also demonstrates the crucial dependences of device performance on the perovskite microstructure in PSCs.

Organic-inorganic hybrid perovskites as promising light harvesting materials have been the focus of scientific research and development of photovoltaics recently^1^,^2^,^3^,^4^,^5^,^6^. Especially, metal halide perovskites (CH_3_NH_3_PbI_3_ and its analogue, normally denoted as CH_3_NH_3_PbI_3−x_Cl_x_) are currently one of the most competitive candidates for the fabrication of solar cells with record breaking efficiencies^7^,^8^, which has been reported to achieve as high as remarkably a certified efficiency of 22.1%^6^,^9^,^10^. However, it is believed that there is still large room to improve the device performance which has been attracting many intensive research efforts worldwide^10^,^11^,^12^,^13^,^14^. One of the fundamental challenges is to obtain good quality perovskite film essentially for high performance perovskite solar cells (PSCs), where charge dissociation efficiency as well as the transport and diffusion lengths of charge carriers are mainly dependent on the crystalline and morphology of these films^13^. The quality of perovskite films is well-known to be highly dependent on their preparation process, such as deposition methods, material composition, and surface properties for the film deposition, the solvents/additives used, and post treatment process. Especially, the post-annealing treatment plays a key role in the formation of high-quality perovskite structure especially using solution process, which directly determines the performance of solar devices^10^,^14^,^15^,^16^,^17^,^18^.

Post-annealing typically serves three main purposes as the following: 1) to remove the residual solvent left from the solution processing; 2) to convert perovskite formation from its precursors, and 3) to enhance crystallization and allow grain growth^19^,^20^,^21^,^22^,^23^. The annealing conditions (such as temperature, time, and atmosphere) as well as the physical characters of the perovskite film (such as its thickness and composition) are known to be critical to the film crystalline, morphology and devices performance^10^,^14^,^15^,^16^,^17^,^18^. For example, Zhou *et al*.^24^ proposed an annealing process in air-heated oven under various humidity environments and found that the more uniform the perovskite film, the better the device performance as well as the performance uniformity. Depending on the precursors used, there are single and mixed halide perovskite films^25^,^26^,^27^,^28^,^29^,^30^,^31^,^32^,^33^,^34^,^35^,^36^,^37^,^38^,^39^. Single halide perovskite film is typically prepared by mixing metal halide and organohalide containing the same halogen (commonly PbI_2_ and CH_3_NH_3_I), which often shows poor quality induced by extensive crystallization caused by solvent evaporation as well as strong ionic interactions between the metalcations and the halides during the annealing treatment process; On the other hand, mixed halide perovskite film is typically prepared using precursors containing more than one halogens (usually PbCl_2_ and CH_3_NH_3_I) which is found to effectively improve the crystalline and morphology of perovskite films due to the removal of excess CH_3_NH_3_^+^ ions with the help of Cl^−^ ions during the post-annealing process^26^,^38^. In comparison with single halide perovskite, mixed halide perovskite typically has to be annealed longer in order to complete the conversion from its precursors into CH_3_NH_3_PbI_3_ perovskite crystal, which would be more thermally stable at room temperature^26^,^38^. However, the post-annealing is proven to be quite tricky to produce high quality film. According to previous reports^18^,^26^,^34^,^35^,^38^, a relatively longer annealing treatment of the mixed halide perovskite at a higher temperature (more than 100 °C) can induce the formation of islands, which is accompanied by the increased content of PbI_2_ phase *via* the loss of CH_3_NH_3_I, resulting in inferior device performance^8^,^26^,^38^. More important, it is often not straightforward to obtain the optimum annealing condition due to the lack of suitable tool to directly monitor the structural evolution conclusively in real time^38^,^39^,^40^. In general, the preparation of perovskite film for solar cell applications nowadays still suffers from poor reproducibility which is largely relied on trial and error. Therefore, it is meaningful and urgent to clarify the role of post-annealing to obtain the optimum condition achieve high performance devices with good reproducibility as well as to disclose the fundamental crystallization mechanism^41^,^42^,^43^,^44^.

X-ray diffraction (XRD) is an ideal structural characterization tool which has been widely used in the study of the perovskite films, for example, to diagnose the formation of the perovskite structure and to track the residual precursors^10^,^18^,^40^,^41^,^42^,^43^,^44^,^45^,^46^,^47^,^48^,^49^,^50^,^51^,^52^,^53^,^54^,^55^. Moreover, *in situ* real-time XRD is able to investigate perovskite thin films during their deposition and post-processing to monitor their phase transition^32^,^33^,^42^. By using *in situ* real-time grazing incidence X-ray diffraction (GIXRD), Saliba *et al*.^31^ found that a short rapid thermal annealing at 130 °C led to the growth of large micron-sized textured perovskite domains and improved the short circuit currents and power conversion efficiencies up to 13.5% for the planar heterojunction perovskite solar cells; Tan *et al*.^33^ also used *in situ* real-time GIXRD and found that there were three distinct structures during the annealing of CH_3_NH_3_PbI_3−x_Cl_x_: a crystalline precursor structure, a 3D perovskite structure, and a mixture of compounds from degradation; Barrows *et al*.^42^ further carried out *in situ* grazing incidence wide angle X-ray scattering (GIWAXS) and grazing incidence small angle X-ray scattering (GISAXS) measurements to characterize and quantify the transition from a precursor crystalline into the perovskite structure during annealing, which revealed device performance evolution of the pristine PEDOT:PSS-based CH_3_NH_3_PbI_3−x_Cl_x_ PSCs with an optimized power conversion efficiency(PCE) up to 12.7%.

Herein, a time-temperature dependent (TTD) post-annealing procedure with an optimized annealing time for perovskite film in a flowing nitrogen atmosphere is demonstrated to be efficient and led to a PCE of 14.36% in the present PEDOT:PSS based PSC in this work, which is one of the highest reported PCE for PSCs using pristine PEDOT:PSS^31^,^32^,^42^,^56^,^57^,^58^. The schematic configuration of the present PSCs with a structure of ITO/PEDOT:PSS/CH_3_NH_3_PbI_3−x_Cl_x_/PC_70_BM/Bphen/Ag is shown in Fig. 1(a). In order to understand how annealing time influence the performance of PSCs, *in situ* real time two dimensional (2D) synchrotron-based GIXRD, which is briefly illustrated in Fig. 1(b), was used to investigate the annealing process of CH_3_NH_3_PbI_3−x_Cl_x_ perovskite thin films, during which a step-by-step gradual transformation was found from distinct mainly organic-inorganic hybrid materials into highly ordered CH_3_NH_3_PbI_3_ crystals. Notably, a re-crystallization process was observed for the first time during annealing, helping to enhance crystallization and prefer-orientation of perovskite crystal, which could explain the drops of the open circuit voltage (V_oc_) and the fill factor (FF) during the ramping of temperature as well as the optimized power conversion efficiency up to 14.36% achieved after an optimum annealing time. The transformation was completed after the same annealing time used for the optimized device and the film started to degrade afterwards with PbI_2_ generation. Thus, the present study not only reveals a vivid picture about the crystallization process, which illustrates clearly the decisive roles of post-annealing in the formation of solution-processed perovskite, but also demonstrates the crucial dependences of device performance on the perovskite microstructure in PSCs.

## Results

### Photovoltaic Performance

**Figure 2:**
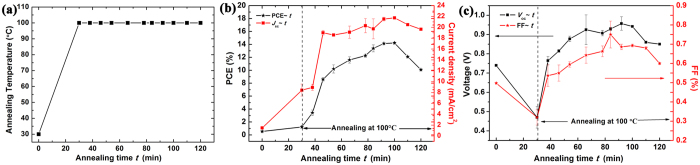
The annealing temperature as the function of ramping time in (**a**); PCE and *J*_*sc*_ of the PSC devices after different annealing time at 100 °C as functions of annealing time in (**b**); *V*_*oc*_ and FF as functions of annealing time in (**c**). Data were collected from 12 devices of each type.

**Figure 3:**
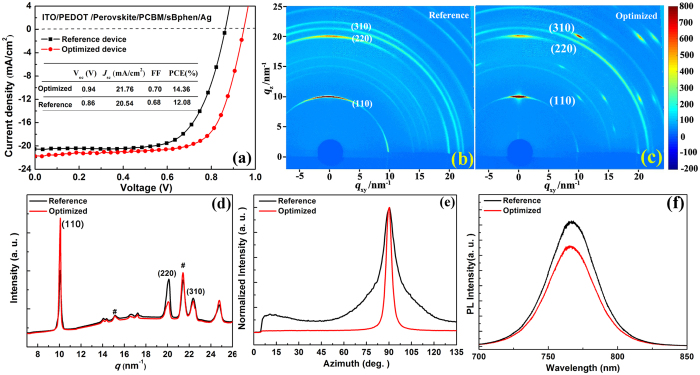
(**a**) *J-V* characteristics of the optimized PSC after annealing for 100 min. at 100 °C and the reference device. The 2D-GIXRD patterns of the reference perovskite film in (**b**) and the optimized perovskite film in (**c**), respectively; (**d**) The azimuthally integrated intensity profiles for the two films derived from (**b**) and (**c**); The peaks at *q* ≈ 10, 20, and 22.4 nm^−1^ denote the signature diffraction peaks (110), (220) and (310) for the perovskite, respectively. The “#” diffraction peaks come from ITO substrate. (**e**) The corresponding radially integrated intensity plots along the ring at *q* ≈ 10 nm^−1^ for the two films derived from (**b**) and (**c**). (**f**) The steady-state PL spectra of the reference and optimized perovskite films measured at room temperature.

To testify the annealing effects on the photovoltaic performance, a series of PSCs were fabricated using perovskite prepared and annealed for different periods of time (0, 30, 38, 46, 66, 78, 92, 100, 110 and 120 min) in a flowing nitrogen atmosphere as described before. Figure 2(a) plots the annealing temperature as the function of ramping time, which is better to the whole annealing process. Their electrical output characteristics are extracted from their measured current-voltage (*J-V*) curves and plotted as functions of annealing time in Fig. 2. It is clear that the PCE and the short circuit current density (*J*_*sc*_) of these devices increase almost continuously with the annealing time till their maxima at 100 min and then decrease at 110 min as shown in Fig. 2(b). Except that they drop clearly during the ramping of temperature, both the open circuit voltage (*V*_*oc*_) and the fill factor (FF) increase gradually with annealing time and saturate as well at 110 min in Fig. 2(c). All the performance characteristics reach their maxima at 100 min should be related to the completion of the structural transformation from precursor materials into high quality perovskite film as reported by Tan *et al*.^31^,^32^,^42^ Fig. 3(a) plots *J-V* curve of the present optimized device after being annealed for 100 min at 100 °C and that of a reference device prepared in the same way except that using the annealing procedure described in our previous reports^30^,^56^,^57^,^58^. Briefly, that annealing procedure was similar to those widely used in many studies by simply increasing the temperature rapidly to 100 **°**C and kept at this temperature for 30 min on a hot plate in a glove box filled with nitrogen. The present optimized device using the TTD annealing procedure in a flowing Nitrogen atmosphere obviously outperform the reference device with a PCE of 14.36%, a FF of 0.7, a *J*_*sc*_ of 21.76 mA/cm^2^, and a *V*_*oc*_ of 0.94 V, which makes it one of the best pristine PEDOT:PSS-based CH_3_NH_3_PbI_3−x_Cl_x_ PSCs reported so far^32^,^33^,^42^,^56^,^57^,^58^. To find out the characteristics of the optimized device as well as to explain the observed evolutions in Fig. 2, the following will turn to GIXRD, Scanning electron microscopy (SEM) and light spectrum measurements.

Figure 3(b) and (c) show the 2D-GIXRD pattern of a reference perovskite film using the annealing procedure described in our previous reports^31^,^56^,^57^,^58^ and that of the film using the TTD annealing procedure for 100 min, respectively. Similar diffraction patterns with bright rings were obtained at *q* ≈ 10, 20, and 22.4 nm^−1^, corresponding to the (110), (220), and (310) crystal planes, respectively, which demonstrates that textured perovskite crystals with an orthorhombic crystal structure^31^,^33^,^34^,^56^,^57^,^58^ are formed in both films. Figure 3(d) reports the azimuthally integrated intensity profiles for the two films derived from Fig. 3(b and c). The strong and sharp (110) peaks in Fig. 3(d) imply that the films possess good crystallization and large crystal size. Compared with the reference, the present film displays a higher and sharper perovskite (110) diffraction peak due to its better quality crystallization^56^, which obviously contribute to the enhanced performances of the present optimized PSCs^31^,^56^,^57^,^58^. It is well known that the crystallographic orientation of the structure domains in all directions can be examined in detail by radially integrating the corresponding scattered ring^22^,^31^,^32^. Figure 3(e) gives these plots along the ring at *q* ≈ 10 nm^−1^ for the two films derived from Fig. 3(b and c). Both the films exhibit a preferential in-plane orientation with their sharp peaks at 90° azimuth angle, however, the present perovskite film shows clearly a much higher orientation order than the reference one, as indicated by its much sharper peak. Thus, the strongly enhanced preferential in-plane orientation also plays a key role to improve performances in the present optimized PSCs^31^,^56^,^57^,^58^. Figure 3(f) reports the steady-state photo-luminescence (PL) spectra of the reference and optimized perovskite films, which are both dominated by the well-known perovskite band-gap emission peak centered at ~760 nm due to the charge carrier recombination^56^,^57^,^58^. It is obvious that this peak of the optimized film is lower than that of the reference, indicating the PL quenching efficiency of this film on hole transport layer (HTL) increased effectively, which is beneficial for hole transport and extraction to ITO electrode and helps to enhance both *J*_sc_ and PCE of the optimized PSC^43^,^56^,^57^,^58^.

### *In situ* real-time GIXRD

**Figure 4:**
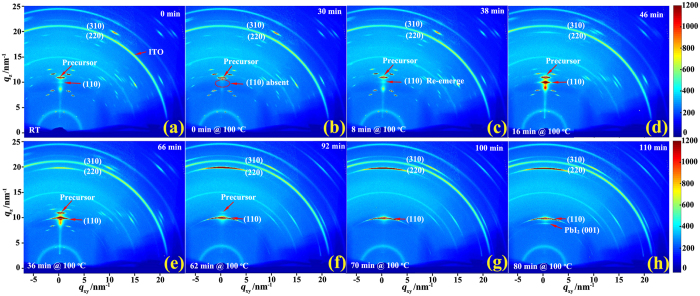
2D-GIXRD patterns of a perovskite film before annealing, after annealing for 30, 38, 46, 66, 92, 100, and 110 min. in (**a**–**h**), respectively.

To study annealing effects in the formation of perovskite crystal structure in CH_3_NH_3_PbI_3−x_Cl_x_ perovskite film, *in situ* real-time 2D-GIXRD were performed by recording the diffraction patterns during annealing process. The TTD annealing process of a perovskite film was closely monitored on a PEDOT: PSS substrate, which started gradually from RT to 100 °C in a dwell of 30 min. (stage I) and remains at 100 °C constantly for 80 min. (stage II). Figure 4 reports the 2D-GIXRD patterns of the film recorded at eight different time points during the annealing process. In Fig. 4(a) for the as-prepared film, there are bright streaks along several weak rings indicating highly textured crystal domains with preferential orientations. Especially, the brightest streak located at *q*_z_ ≈ 11 nm^−1^ along the out-of-plane direction is ascribed to the crystalized mixture of CH_3_NH_3_I and PbCl_2_, which is also denoted as precursor structure by Tan *et al*.^32^,^33^,^42^ or as CH_3_NH_3_PbCl_3_ structure reported by Yang *et al*.^10^ At *q*_z_ ≈ 8.6 nm^−1^ along the out-of-plane direction, a less bright mysterious spot is also visible, which is not from CH_3_NH_3_I, PbCl_2_, PbI_2_, neither perovskite^42^. It is also noticed that there is another weak spot at *q*_z_ ≈ 10 nm^−1^, which is known to be due to tetragonal perovskite (110) diffraction^8^,^10^,^22^,^31^. This observation of weak perovskite (110) diffraction peak indicates that trace amount of perovskite is already formed just after spin-coating without any post-annealing. Thus, the as-prepared film is dominated by precursor structures with only trace amount of perovskite formed. It is rather interesting that the perovskite peak at *q*_z_ ≈ 10 nm^−1^ disappears at 30 min when temperature reaches 100 °C in Fig. 4(b), which indicates that the pristine perovskite structure becomes disordered during the ramping of temperature. It is noticed that the mysterious peak at *q*_z_ ≈ 8.6 nm^−1^ is absent as well, even though the two streaks at two sides of this peak with the same *q*_r_ ≈ 8.6 nm^−1^ are visible from Fig. 4(a) to (e). Just after 8 min. at 100 °C, it is clear that the peak at *q*_z_ ≈ 10 nm^−1^ and that at *q*_z_ ≈ 8.6 nm^−1^ resurfaces with almost unchanged bright precursor streak at *q*_z_ ≈ 11 nm^−1^ in Fig. 4(c). Afterwards, the intensity of perovskite (110) peak grows with annealing time, which becomes only slightly dimmer than the precursor peak at 46 min. in Fig. 4(d) and then more bright at 66 min. in Fig. 4(e), respectively. The comparable diffraction intensities of the perovskite and precursor peaks in Fig. 4(d) and (e) indicate coexistence of these two structures with comparable amounts. It is interesting that the mysterious peak is enhanced at 46 min. in Fig. 4(d) but becomes weaker again at 66 min. in Fig. 4(e). At 92 min in Fig. 4(f), the perovskite diffraction (110) peak becomes dominant whereas that precursor peak becomes dim and the mysterious peak disappears. Eventually, the perovskite film is optimized at 100 min with only all the sharp and bright perovskite features but without the precursor peak as shown in Fig. 4(g), even though the temperature is still 100 °C. It is also noticed that the perovskite (220, 310) sharp scattering rings gradually appear and become well resolved during annealing as shown in Fig. 4(a–g), indicating highly ordered crystallinity formation after annealing for 100 min^22^,^31^,^32^. Even longer annealing causes a new diffraction streak attributed to PbI_2_ (001) peak to appear at *q* ≈ 9 nm^−1^ in Fig. 4(h), indicating decomposition of perovskite starts.

From these *in-situ* GIXRD results, it becomes clear that the transformation from distinct mainly organic-inorganic hybrid materials into highly ordered CH_3_NH_3_PbI_3_ crystal occurs gradually during the post-annealing process of the solution-processed CH_3_NH_3_PbI_3−x_Cl_x_ perovskite film, which is well consistent with the reports by Saliba *et al*.^32^, Tan *et al*.^33^, and Alexander *et al*.^42^ Too short or too long annealing time would lead to either incomplete transformation with unconverted precursors or decomposition of perovskite, respectively. This immediately means that it is necessary to anneal the film for an optimum time to complete this transformation, which is consistent with their device performance evolution. Taking into account of the disappearance of perovskite structure during the ramping of temperature, the formation of perovskite structure should involve a re-crystallization process during annealing, which is not observed in the similar reports by Saliba *et al*.^32^, Tan *et al*.^33^, and Barrows *et al*.^42^ The re-crystallization process of perovskite structure might be related with chlorine-ion rich concentrating in the surface layer of CH_3_NH_3_PbI_3−x_Cl_x_ perovskite film, which has been reported by Colella *et al*.^40^ and Tripathi *et al*.^55^ The observed re-crystallization should be the main reason for the decreases of the V_oc_ and FF in the PSCs during the ramping of temperature and then their gradual increases with annealing time afterwards in Fig. 2(c). As this process could also help to enhance crystallization and preferential orientations of perovskite crystal, a higher PCE up to 14.36% was thus achieved using such an annealing procedure than those in the similar reports^32^,^33^,^56^,^57^,^58^.

**Figure 5:**
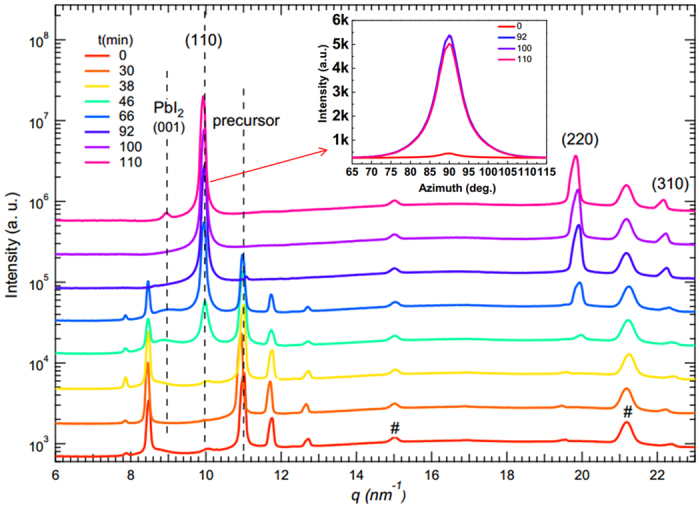
The azimuthally integrated intensity profiles derived from the GIXRD patterns in Fig. 4. The dotted lines at *q* ≈ 9, 10, and 11 nm^−1^ denote the characteristic diffraction peaks of PbI_2_ (001), perovskite (110), and precursor crystalline structure, respectively. The “#” diffraction peaks come from ITO substrate. The insert is the corresponding radially integrated intensity of the diffraction rings at *q* ≈ 10 nm^−1^ at four time points (0, 92, 100, 110 min).

Figure 5 plots the azimuthally integrated intensity profiles derived from the GIXRD patterns in Fig. 4, which shows quantitatively the gradual structural evolution from distinct mainly precursors into highly ordered CH_3_NH_3_PbI_3_ crystal. It is clear that the precursor peak at *q* ≈ 11 nm^−1^ gradually decreases whereas the initial tiny perovskite (110) peak at *q* ≈ 10 nm^−1^ disappears at 30 min, re-emerges at 38 min, and then keeps increasing till it becomes dominant around 100 min. Thus, Fig. 5 further supports a gradual transformation and a re-crystallization of perovskite structure during annealing to form highly ordered perovskite. In Fig. 5, it is interesting that the peak at *q* ≈ 8.6 nm^−1^ exists at the beginning and disappears after 92 min similarly as the precursor peak during the annealing process. As mentioned before in Fig. 4(b), the mysterious peak at *q*_*z*_ ≈ 8.6 nm^−1^ along the out-of-plane direction disappears together with the perovskite peak. These two facts indicate the origin of the peak with *q*_*z*_ ≈ 8.6 nm^−1^ as mixed structures of pervoskite and the precursor phase with different preferential directions. Thus, these observations suggest that the gradual structural transformation to form pervoskite is quite complicated during annealing.

In the insert of Fig. 5, the radially integrated intensity plots along the ring corresponding to the typical (110) perovskite crystalline plane at *q*_z_ ≈ 10 nm^−1^ for the film at four different time points are plotted as functions of azimuth angle. The comparison of these plots shows that the (110) planes in the film during annealing always display a preferential in-plane orientation leading to the sharpest peaks at an azimuth angle of 90°. Moreover, it is obvious that the peak height reaches its maximum at 100 min, indicating a highly ordered perovskite structure formed with a preferential in-plane orientation^8^,^15^,^31^.

Thus, the present *in situ* real time 2D-GIXRD obviously provides rich and useful information elucidating perovskite formation dynamics during annealing. Precursor crystalline structure is dominant with only trace amount of perovskite crystallines and the complicated transition from precursor materials into perovskite crystallines occurs gradually during the annealing process, which supports Ostwald’s ‘Rule of Stages’^51^ in which a metastable precursor structure is first formed, followed by transformation into the more stable perovskite crystal structure. These interesting results actually reflect the importance of the real time structural study which builds up a clear relationship between perovskite structure and its device performance. After being annealed for the optimum time, the formed highly ordered in-plane perovskite structure enables more uniform charge generation and collection as well as reduces the leakage with fewer shunt paths, which enhances the device performance of PSCs^19^,^31^,^42^,^45^,^55^,^56^,^57^,^58^.

**Figure 6:**
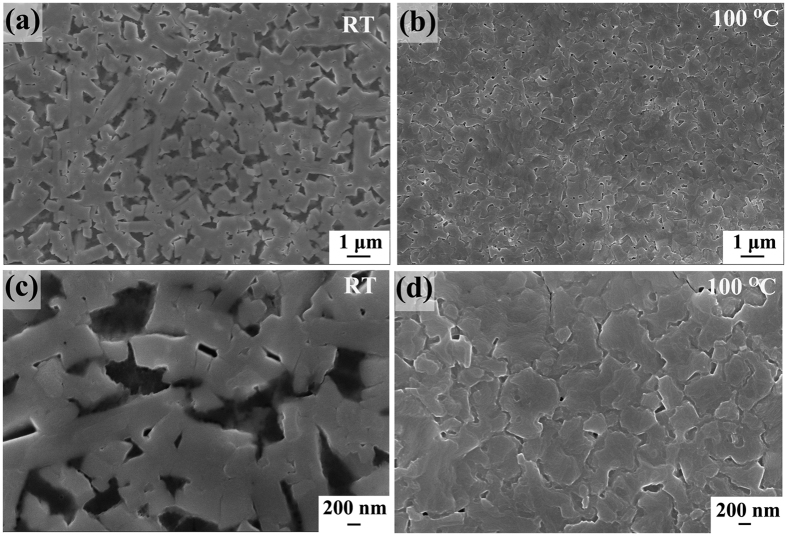
SEM images of perovskite films on PEDOT: PSS before and after being annealed for the optimum time in (**a**–**d**) respectively.

The formation of high quality film after the optimum annealing time is also in line with SEM results as shown in Fig. 6, where the annealing-free film displays elongated islands separated by large pores/gaps (often around several hundred nanometers wide) in Fig. 6(a) and (c), whereas the film after being annealed for the optimum time exhibits large textured domains with a nearly complete coverage in Fig. 6(b) and (d). While the worse morphology of the annealing-free film with large voids leads to losses in device efficiency, the high surface coverage of the annealed film enhances the device performances^56^,^57^,^58^.

From the present study, it can be concluded that annealing of the as-prepared perovskite films for an optimum time is crucial for high performance PSCs on three aspects: 1) to achieve high crystal quality with prefer orientations; 2) to increase film uniformity with high surface coverage; 3) to enhance optical absorption. As shown in previous reports, varying preparation procedures will affect the crystallization and crystal orientation of the perovskite film greatly^15^,^22^,^25^,^31^,^32^,^33^,^39^,^40^,^41^,^42^,^43^,^44^,^56^,^57^,^58^, the present study unambiguously demonstrates the decisive roles of post-annealing in the formation of high quality solution-processed perovskite, which are proven to be crucial for the photovoltaic applications.

In the present study, a re-crystallization process during annealing is identified. The annealing induced formation of perovskite structure is not a simple perovskite crystallization and grain growth process. There are two processes going on during annealing: the first is a gradual transition from distinct mainly precursor crystalline structures into perovskite structures^32^,^33^,^34^; the second is perovskite structures nucleate to form grains which grow into large crystalline eventually^44^,^55^. The few pristine perovskite structures formed before annealing are randomly distributed in the majority precursor crystalline structures. During ramping temperature, the temperature is not high enough and time is too short to trigger sufficient chemical transformation to provide enough extra perovskite structures to grow perovskite crystal grains but to enhance the crystallization of precursor crystalline structures, which is supported by the intensity of the precursor peaks increase at this stage. In the same time due to such a high temperature, the few perovskite pristine structures could easily disperse into the surrounded precursors and become disordered, which causes the disappearance of the diffraction peaks of the perovskite. After some time at 100 °C, the chemical transformation gradually becomes intensified leading to more and more perovskite structures formed, which eventually nucleate to form perovskite grains and then grow into large perovskite crystalline with increasing time. The pristine perovskite structures randomly dispersed in the film at ramping temperature could serve well as uniformly distributed nucleation center for the crystallization process, which would help enhance the formation of the high quality perovskite structure.

## Discussions

In this work, how annealing step-by-step determines the formation of CH_3_NH_3_PbI_3_ perovskite structure and thus their devices performance is investigated in details by *in situ* GIXRD. In the post-annealing process, as-prepared films undergo a gradual transition from distinct mainly precursor crystalline structures into highly ordered perovskite structure whereas the few pristine structure actually becomes disordered with ramping temperature indicating a re-crystallization process during annealing not reported previously. It is also proved the sensitive relationship between the evolution of perovskite structure and the distinctly photovoltaic parameters of PSCs. Thus, a clear understanding of the transformation from organic-inorganic hybrid materials into CH_3_NH_3_PbI_3_ perovskite is established which facilitates the production of high-quality perovskite films for the development of high performance solar cells. Especially, the optimization of a well-controlled annealing procedure is shown to be crucial to fabricate high quality perovskite film for high performance PSCs.

## Methods

### Materials and sample preparation

Methylammonium iodide (MAI) was synthesized following a one-step method reported in literature^27^. Bphen was purchased from Nichem Fine Technology Co. Ltd. (Taiwan). PCBM and PEDOT:PSS (CLEVIOS Al 4083) were purchased from Solenne and Heraeus, respectively.

To prepare the perovskite precursor solution, MAI and lead chloride (PbCl_2_, 99.999%, Alfa) powder were mixed in anhydrous DMF (amine free; 99.9%, Aldrich) with a molar ratio of 3:1. The perovskite precursor solution was stirred at 70 °C overnight and then filtered through PTFE filters (0.22 μm) before use.

### Fabrication of inverted planar PSCs

The glass substrates coated with patterned indium tin oxide (ITO) with a sheet resistance of around 10Ω·s·q^−1^ were first cleaned with detergent, followed by ultrasonic cleaning in acetone and ethanol, and were then dried by blowing nitrogen. Subsequently, substrates were treated by ultraviolet ozone plasma for 15 min. PEDOT:PSS aqueous solution was spin-coated onto the substrate at 4500 r.p.m. for 60 s. After being baked at 120 °C for 20 min, substrates were then transferred into a nitrogen-filled glove box, where 30 wt% (weight percentage) perovskite precursor solution was spin-coated at 4000 r.p.m. for 40 s. After laid on the petri dish at room temperature (RT) for about 15 min, the perovskite films were then annealed using a TTD procedure as described in the following in a sealed cell filled with nitrogen kept continuously flowing at a steady speed of 0.05 L/min: heated to 100 °C from RT over a dwell of 30 min, kept at 100 °C for different periods of time, and cooled down to RT in another 20 minutes. The PCBM layers were then spin-coated above the perovskite films using 20 mg/mL chlorobenzene solution at 2000 r.p.m for 40 s, and subsequently a bephene interfacial layer was then spin-coated^46^. Finally, a 100-nm-thick Ag cathode layer was evaporated through a shadow mask in a chamber with a base pressure of 10^−6^ Torr (Minispectra, Kurt J. Lesker) installed in the same glove box. The device active area was estimated to be around 7.25 mm^2^.

### Devices characterization

Device characteristics, such as the typical current density-voltage (J–V) curves, were measured in a nitrogen-filled glovebox under a Newport 94023 A solar simulator as irradiation source, which was equipped with a 300 W Xenon lamp and an air mass (AM) 1.5 G filter to generate simulated AM 1.5 G solar spectrum. The irradiation intensity was 100 mW/cm^2^ calibrated by a Newport standard silicon solar cell 91150.

### Perovskite film characterization

UV-Visible absorption spectrum of the perovskite films on PEDOT:PSS-coated ITO glasses were measured using an UV/Vis spectrophotometer (PerkinElmer Lambda 750). Steady-state photo-luminescence (PL) measurements were recorded by an Edinburgh Instruments FLS920 fluorescence spectrometer equipped with a 532 nm pulsed laser as an excitation source. Scanning electron microscopy (SEM) images were obtained by a field emission scanning electron microscope (FEI Quanta 200), while the grazing incidence X-ray diffraction (GIXRD) measurements were performed at the BL14B1 beamline of Shanghai Synchrotron Radiation Facility (SSRF)^46^,^47^,^48^,^49^. The two dimensional GIXRD (2D-GIXRD) patterns were acquired by a MarCCD detector mounted vertically at a distance of around 223 mm from the sample with an exposure time of less than 20 sec and a grazing incidence angle of 0.2° with respect to the surface plane. The 2D-GIXRD patterns were analyzed afterwards using the Fit 2D software and displayed in scattering vector *q* coordinates with *q* = 4πsinθ/λ, where θ is half of the diffraction angle, and λ is the wavelength of incident X-ray^49^.

## Additional Information

**How to cite this article:** Yang, Y. *et al*. Annealing Induced Re-crystallization in CH_3_NH_3_PbI_3−x_Cl_x_ for High Performance Perovskite Solar Cells. *Sci. Rep.* **7**, 46724; doi: 10.1038/srep46724 (2017).

**Publisher's note:** Springer Nature remains neutral with regard to jurisdictional claims in published maps and institutional affiliations.

## Figures and Tables

**Figure 1:**
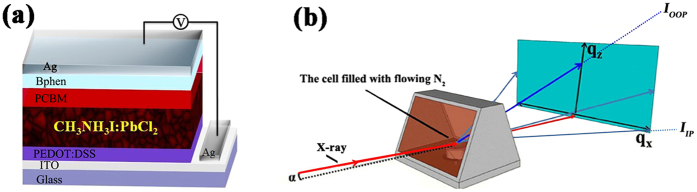
(**a**) Schematic of the perovskite solar cells with a structure of ITO/PEDOT:PSS/CH_3_NH_3_PbI_3−x_Cl_x_/PC_70_BM/Bphen/Ag. (**b**) The GIXRD experimental setup, where *a, I*_OOP_, *I*_IP_, is the beam incident angle, the intensity of scattering beam along the out-of-plane (OOP) and in-pane (IP) directions, respectively. The samples are in a sealed cell with flowing nitrogen (N_2_).
